# Mobile Antibiotic Resistance Encoding Elements Promote Their Own Diversity

**DOI:** 10.1371/journal.pgen.1000775

**Published:** 2009-12-18

**Authors:** Geneviève Garriss, Matthew K. Waldor, Vincent Burrus

**Affiliations:** 1 Centre d'Étude et de Valorisation de la Diversité Microbienne (CEVDM), Département de biologie, Université de Sherbrooke, Sherbrooke, Québec, Canada; 2 Channing Laboratory, Brigham and Women's Hospital and Harvard Medical School, Boston, Massachusetts, United States of America; 3 Howard Hughes Medical Institute, Boston, Massachusetts, United States of America; Baylor College of Medicine, United States of America

## Abstract

Integrating conjugative elements (ICEs) are a class of bacterial mobile genetic elements that disseminate via conjugation and then integrate into the host cell genome. The SXT/R391 family of ICEs consists of more than 30 different elements that all share the same integration site in the host chromosome but often encode distinct properties. These elements contribute to the spread of antibiotic resistance genes in several gram-negative bacteria including *Vibrio cholerae*, the agent of cholera. Here, using comparative analyses of the genomes of several SXT/R391 ICEs, we found evidence that the genomes of these elements have been shaped by inter–ICE recombination. We developed a high throughput semi-quantitative method to explore the genetic determinants involved in hybrid ICE formation. Recombinant ICE formation proved to be relatively frequent, and to depend on host (*recA*) and ICE (*s065* and *s066*) loci, which can independently and potentially cooperatively mediate hybrid ICE formation. *s065* and *s066*, which are found in all SXT/R391 ICEs, are orthologues of the bacteriophage λ Red recombination genes *bet* and *exo*, and the *s065*/*s066* recombination system is the first Red-like recombination pathway to be described in a conjugative element. Neither ICE excision nor conjugative transfer proved to be essential for generation of hybrid ICEs. Instead conjugation facilitates the segregation of hybrids and could provide a means to select for functional recombinant ICEs containing novel combinations of genes conferring resistance to antibiotics. Thus, ICEs promote their own diversity and can yield novel mobile elements capable of disseminating new combinations of antibiotic resistance genes.

## Author Summary

Integrating and conjugative elements (ICEs) are a class of mobile elements found in diverse bacteria. ICEs of the SXT/R391 family have enabled the dissemination of genes conferring resistance to antibiotics among several important pathogens, including *Vibrio cholerae*, the agent of cholera. Here, using comparative analyses of the genomes of several SXT/R391 ICEs, we found that these elements are mosaics that have been shaped by inter–ICE recombination. We developed a plate-based method for semi-quantitative analyses of the genetic requirements for hybrid ICE formation. We discovered that hybrids form at relatively high frequencies and that both host and ICE genes can function independently and potentially cooperatively to mediate hybrid formation. The ICE–encoded recombination genes, which are found in all SXT/R391 ICEs, are related to genes that mediate recombination in bacteriophages, but have not been described previously in conjugative elements. Conjugative ICE transfer was not required for hybrid ICE formation but facilitates the segregation of hybrids. Thus, ICEs promote their own diversity and the generation of recombinant ICEs can yield novel mobile elements capable of disseminating new combinations of antibiotic resistance genes.

## Introduction

Mobile genetic elements, including bacteriophages, conjugative plasmids and integrating conjugative elements (ICEs), are key mediators of bacterial genome evolution [1]. These elements can rapidly spread in bacterial populations and often confer to host bacteria selectable traits that are advantageous in particular environments or enable adaptation to new ecological niches. Transfer of ICEs and plasmids from donor to recipient bacteria occurs via conjugation, a process that requires direct cell-to-cell contact [2],[3]. Conjugative transmission of ICEs and plasmids has limited the clinical usefulness of many antibiotics, since these mobile elements are potent vectors for dissemination of antibiotic resistance genes in bacterial populations [2], [4]–[7].

ICEs integrate into and replicate along with the host cell chromosome, whereas plasmids exist as extra-chromosomal (usually circular) autonomously replicating DNA molecules. ICEs can excise from the donor cell chromosome and form circular molecules that are thought to be the substrates for the conjugative machinery. Similar to most conjugative plasmids [8], ICE conjugative DNA transfer is thought to be initiated at a specific *cis-*acting site (*oriT*) required for efficient translocation of the DNA to the recipient cell through the mating bridge. Within the recipient cell, host enzymes are thought to convert the translocated single-stranded DNA into double-stranded DNA that is circularized. An element-encoded recombinase (integrase) enables the integration of the ICE into the chromosome of the new host [2], [9]–[11].

ICEs are widespread among diverse taxonomic groups of bacterial species and are able to transfer between genetically unrelated bacteria [5], [10]–[12]. The SXT/R391 family of ICEs, which is one of the largest and most diverse set of ICEs studied, includes elements that have been detected in clinical and environmental isolates of several species of γ-proteobacteria from four continents over the past 40 years [13]–[20]. In Asia and Africa, this family of ICEs has played an important role in the spread of genes conferring resistance to multiple antibiotics in *Vibrio cholerae*, the causative agent of cholera [17], [19], [21]–[23]. Currently, nearly all isolates of *V. cholerae* from cholera patients from these two continents harbor SXT, a prototypical member of the SXT/R391 family originally isolated from a 1992 Indian *V. cholerae* O139 isolate, or a closely related ICE [17]–[19], [24]–[26].

**Figure 1:**
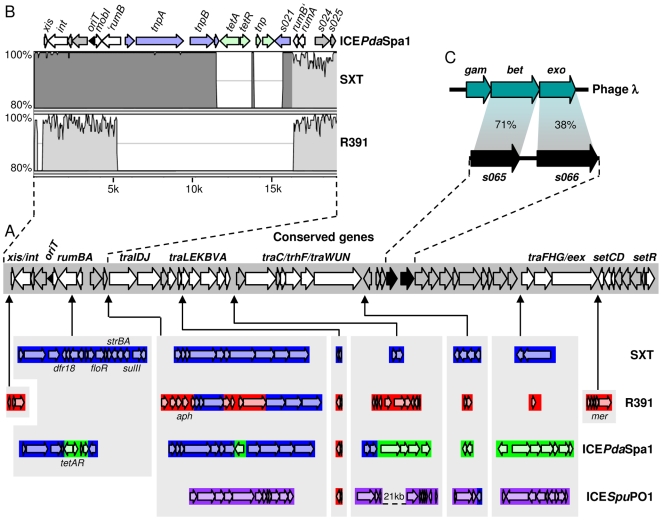
Evidence suggesting that recombination occurs between SXT/R391 ICEs. (A) The middle gray box represents the set of genes (arrows) conserved in the 4 SXT/R391 genomes shown. Gray ORFs represent genes of unknown function, white ORFs represent genes of known function [28],[52],[53], and black ORFs correspond to *s065* and *s066*. Below, variable ICE regions are shown with colors according to the elements in which they were originally described: SXT [28] (blue), R391 [29] (red), ICE*Pda*Spa1 [15] (green), and ICE*Spu*PO1 [30] (purple). (B) A close-up of the *attL*-*s025* region of ICE*Pda*Spa1 (accession number AJ870986) is shown in the upper left. The variation of percentage of identity was plotted using a Multi-LAGAN pairwise comparison [63] of this ICE*Pda*Spa1 region with the corresponding regions of SXT (accession number AY055428) and R391 (accession number AY090559) and the mVista visualization module [64] with a sliding window of 100 bp. The minimum width and the minimum percent conservation identity that must be maintained over that width for a region to be considered conserved were set at 100 bp and 70% respectively. The dark gray area highlights the large nearly identical region conserved between SXT and ICE*Pda*Spa1. (C) A comparison of *s065* and *s066*, which are present in all SXT/R391 ICEs, to the bacteriophage λ Red genes (numbers represent % similarity between S065 and Bet, and S066 and Exo, respectively) is shown in the upper right. *drf18* encodes trimethoprim resistance; *floR* encodes chloramphenicol resistance; *strAB* encodes streptomycin resistance; *sulII* encodes sulfamethoxazole resistance; *tetAR* encodes tetracycline resistance; *aph* encodes kanamycin resistance; and *mer* encodes mercury resistance.

The ICEs of the SXT/R391 family are grouped together because they all encode a highly conserved integrase (Int) that mediates the elements' site-specific integration into the host genome in the 5′ end of *prfC*, a conserved gene encoding the peptide chain release factor RF3 [27]. Based on knowledge of the ∼100-kb genomes of several SXT/R391 ICEs [15], [28]–[31], in addition to the conserved integrase gene (*int*), these elements all contain a conserved set of ∼24 genes that mediate their common functions that include: excision/integration, conjugative transfer and regulation [5]. Distinct variable regions that confer element-specific phenotypes, such as synthesis of the second messenger c-di-GMP or resistance to antibiotics or heavy metals are interspersed within this conserved and syntenous SXT/R391 backbone (see Figure 1A) [5],[15],[22],[30],[32].

In some cases, SXT/R391 ICEs do not exclude one another and can be present in the same host [33]–[35], providing the opportunity for the generation of recombinant ICEs. For example, R391, the other prototypical member of the SXT/R391 family, which was originally derived from a 1967 South African *Providencia rettgeri* isolate, and SXT can reside together in the same host [33]. A cell that contains one of these two ICEs can acquire a copy of the other ICE, yielding tandem arrangements of SXT and R391 in the host chromosome [33]. Tandem repeat structures are often excellent substrates for recombination [36] and exconjugants derived from donor strains containing such tandem arrays sometimes contain hybrid ICEs with genes from both R391 and SXT [37].

The molecular mechanisms that enable the formation of hybrid ICEs, which may contain novel combinations of genes conferring resistance to antibiotics, have not been addressed. However, two genes, *s065* and *s066*, which are highly conserved (≥96% identity) among all known SXT/R391 ICEs could contribute to the formation of hybrid ICEs. These genes encode proteins that are similar to the recombinase Bet (71% similarity and 55% identity) and the double-strand specific 5′ to 3′ exonuclease Exo (38% similarity and 26% identity) that are encoded by the temperate bacteriophage λ and several other phages [38] (Figure 1C). In λ, Bet and Exo, along with the Gam protein constitute an efficient *recA*-independent recombination system known as λ Red. Classic studies by Stahl and colleagues revealed many of the key features of the λ Red recombination system. They showed that efficient Red-mediated homologous recombination between λ chromosomes was almost entirely dependent on DNA replication [39], which generates a significant population of λ DNA with double-stranded breaks that serve as substrates for Red. Using replication-blocked crosses of phage λ chromosomes containing a single double-stranded cut, Stahl et al proposed that λ Red mediates recombination by a strand annealing mechanism [40]. Red Exo degrades 5′ ends of linear double-stranded DNA, creating 3′ single-stranded overhangs that can serve as templates for Red Bet to pair with complementary single-stranded DNA targets [41]. Red Gam (for which there is no SXT-encoded homologue) inactivates the *E. coli* exonuclease V (RecBCD), thereby protecting the ends of linear double-stranded DNA from degradation [41],[42]. Besides providing significant amounts of double-stranded breaks, replication also provides a single-stranded DNA target for strand annealing on the lagging strand that is exposed by a passing replication fork [43]. Ordinarily, λ recombination is RecA-independent; however, when DNA replication is blocked, λ Red can also mediate efficient recombination via a strand invasion mechanism that is dependent upon RecA function [40],[44]. Poteete et al suggested that the strand invasion pathway is a RecA-dependent salvage pathway for aborted Red-mediated recombination [45]. In recent years, the λ Red system has proven to be extremely useful for genetic engineering of *Escherichia coli* and closely related species [46]–[49]; however, investigation of the function of the Red pathway in its natural context, cells undergoing the λ lytic cycle, has several technical challenges [48]. To our knowledge, λ Red-like recombination systems have not been described previously in conjugative elements.

Here, we found that the genomes of SXT/R391 ICEs appear to be routinely shaped by inter-ICE recombination. We explored the role of the SXT and R391 *bet* and *exo* homologues (*s065* and *s066*) and that of *recA*, a key host recombination gene, in the formation of hybrid ICEs. To accomplish this, we created a high throughput semi-quantitative screening assay that enabled the visual identification of exconjugant colonies containing hybrid ICEs. We found that *recA* mediated the formation of the majority of hybrid ICEs. Both *s065* and *s066* also contribute to the formation of hybrid ICEs and in the absence of *recA*, *s065* and *s066* appear to mediate the formation of nearly all hybrid ICEs. Conjugation was not essential for the formation of hybrid ICEs, suggesting that conjugative transfer acts as a means to segregate hybrid elements into new host cells. Thus, both host- and element-encoded recombination systems promote the formation of the mosaic genomes of SXT/R391 ICEs.

## Results

### Evidence for recombination between SXT/R391 ICEs

When the genomes of SXT [28] and R391 [29] were originally reported, it appeared that the variable regions in this family of ICEs (shown as colored bars underneath the set of shared genes within the gray rectangle in Figure 1A) were element-specific [50]. However, examination of the growing number of sequenced SXT/R391 ICE genomes suggests that even though some variable regions may be element-specific, others are shared by two or more ICEs (e.g. see ICE*Pda*Spa1 and ICE*Spu*PO1 in Figure 1A), suggesting that this family of ICEs undergoes recombination. Closer analysis of conserved regions of these elements also suggested that recombination between SXT/R391 ICEs has shaped their genomes. Pairwise alignments of the genome sequence of ICE*Pda*Spa1, an ICE derived from the fish pathogen *Photobacterium damselae* subsp. *piscicida,* with that of SXT or R391 revealed that the majority of conserved sequences are only 95–97% identical, but that the 11.5-kb *attL*-*tnpB* and 0.6-kb *s021*-*rumB*' regions of ICE*Pda*Spa1 and SXT are nearly 100% identical (Figure 1B). These comparisons suggest that a relatively recent recombination event within the 5′ end of the truncated copy of *rumB*' occurred between precursors of ICE*Pda*Spa1 and SXT, and support the idea that SXT/R391 ICE genomes are mosaics that have been sculpted by inter-ICE recombination. Exchange of DNA segments between these ICEs occurs when these elements are present in the same host cell. The tandem arrays that these ICEs can form in the host chromosome likely provide a suitable substrate for such recombination events to occur.

### Detection of hybrid ICE formation

**Figure 2:**
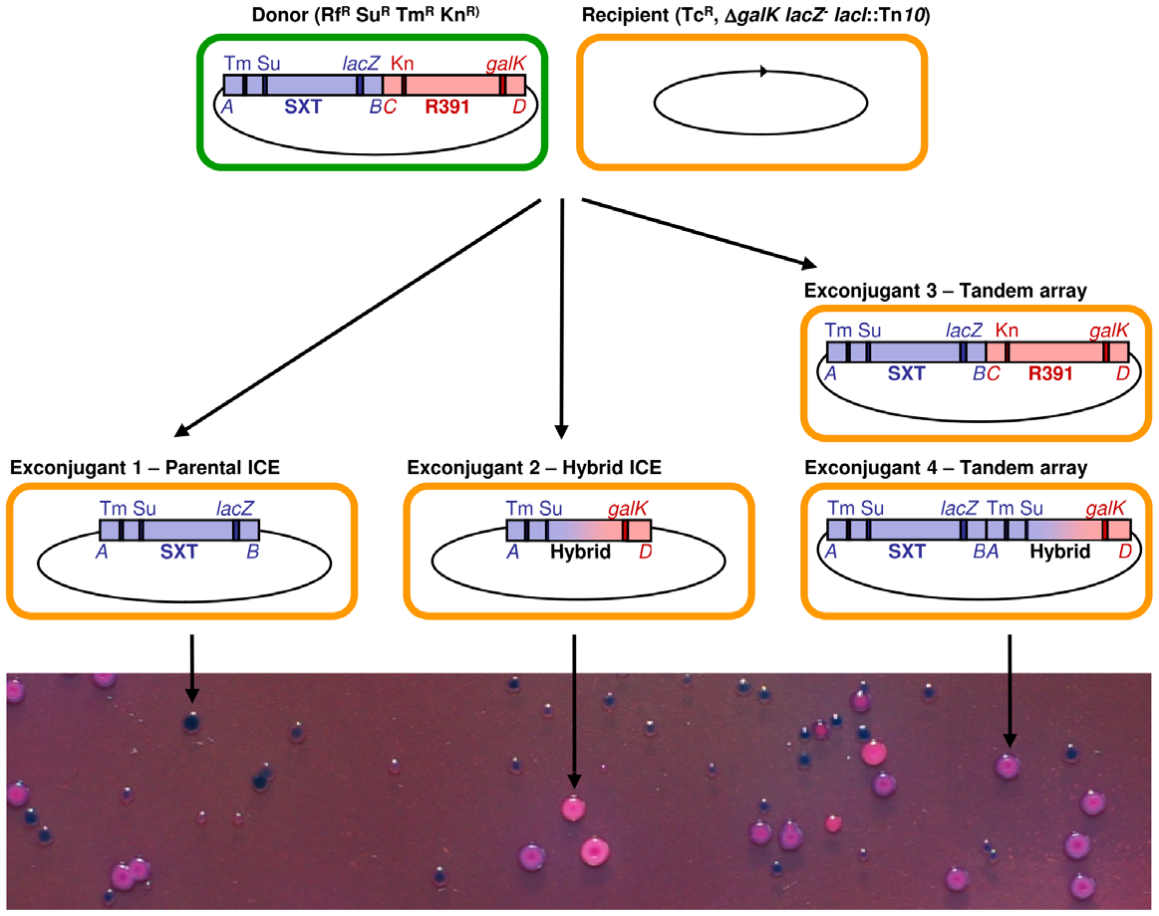
Schematic of colony color-based semi-quantitative assay for the detection of hybrid ICE–containing colonies. Relative positions of resistance markers (trimethoprim (Tm), sulfamethoxazole (Su), kanamycin (Kn)) and phenotypic markers (*lacZ* and *galK*) in SXT and R391 are indicated. DNA originating from SXT is shown in blue and DNA originating from R391 is shown in red. The use of a Δ*galK lacZ* ^−^ *lacI*::Tn*10* recipient strain allows constitutive expression of the inserted *lacZ* and *galK* from the ICEs in the exconjugant colonies. Mating between a donor cell (green) containing an SXT-R391 tandem array and a recipient cell (orange) yields exconjugants that may contain a single element, a hybrid element or a tandem array. MacConkey X-gal D-galactose indicator agar containing trimethoprim, sulfamethoxazole and tetracycline (bottom panel) reveals colonies harboring single parental ICEs (blue colonies), hybrid ICEs (red colonies), and SXT-R391 tandem arrays (purple colonies). Purple colonies may also consist of cells containing an array composed of SXT and a hybrid element on this media (e.g. exconjugant 4). Red and purple colonies are larger on this medium because they can use D-galactose as a carbon source. (A,B) SXT left and right extremities; (C,D) R391 left and right extremities, respectively amplified by primer pairs VISLF/VISLR3, VISRF/VISRR, VISLF/VISLR2, VISRF/VISRR2 [37].

We developed a high throughput conjugation-based semi-quantitative screen to assess the genetic requirements for the formation of hybrid ICEs. The assay employs donor cells bearing tandem copies of modified SXT and R391 and was designed to distinguish between exconjugant colonies containing SXT-R391 tandem arrays, hybrid elements or single parental elements (Figure 2). The phenotypic markers *lacZ* and *galK* were inserted between *traG* and *eex* in SXT and between *traG* and *merR* in R391, respectively (Figure 1A and Figure 2). The position of these two loci, near the right ends of the elements, is remote from the antibiotic resistance markers that are found near the left ends of SXT (*sulII dfr18*) and R391 (*aph*) (Figure 1A and Figure 2), thereby maximizing the opportunity to detect recombination events occurring within tandem arrays. Both *lacZ* and *galK* were placed under control of the P*_lac_* promoter to enable high-level β-galactosidase and galactokinase activities in a *lacI* background. *Escherichia coli* strains containing tandem arrays of these labeled ICEs were used as donors in mating assays using Δ*galK lacZU118 lacI42*::Tn*10* derivatives of *E. coli* MG1655 as recipient strains. Exconjugants were isolated on MacConkey indicator agar plates supplemented with galactose and X-Gal (MCGX) along with the antibiotics sulfamethoxazole (Su) and trimethoprim (Tm) to select for SXT or kanamycin (Kn) to select for R391. Using this medium, we expected to infer the ICE content of each exconjugant colony from its color and resistance phenotypes (e.g., Figure 2), and to thereby determine the percentage of exconjugants containing hybrid elements. PCR assays confirmed our expectations regarding the presence of hybrid ICEs in red colonies on Su-Tm medium and blue colonies on Kn medium (Figure 2 and data not shown). However, PCR analyses also revealed that a subset of purple exconjugant colonies contained tandem arrays consisting of a hybrid ICE coupled to a parental ICE. Thus, our method for enumeration of recombinant ICEs formed in these assays (e.g. as red colonies in Figure 2) understates the true frequency of recombination events.

In pilot experiments, we found that the percentage of hybrid ICEs detected was influenced by which ICE's antibiotic resistance markers were selected. A higher percentage of exconjugants harboring a hybrid ICE was isolated on Su-Tm (6.75%) than on Kn (2.70%). This is probably a consequence of the fact that the R391 transfer frequency is about 10-fold higher than that of SXT, and hence a high frequency of colonies containing hybrids are likely to contain R391 as well, and thus cannot be distinguished from strains containing tandem arrays (39.2% tandem arrays on Su-Tm vs 10.4% on Kn). Consequently, in most subsequent studies of the genetic requirements for hybrid ICE formation, we used donors harboring SXT-R391 arrays and Su Tm to select for hybrid-harboring exconjugants; however, in some experiments we were unable to obtain SXT-R391 arrays with the desired deletions and in these cases we used donors containing R391-SXT arrays.

### *recA* enables the formation of most, but not all, hybrid ICEs

We suspected that the host *recA* gene might play a key role in the generation of hybrid ICEs since the SXT and R391 genomes have more than 95% identity over nearly 64 kb of DNA distributed in 11 segments ranging from 247 bp to 12,085 bp. Hybrid ICEs could form by RecA-mediated homologous recombination either in the donor cells prior to transfer or in recipient cells after transfer of both SXT and R391 from donor cells. We carried out conjugation experiments using *recA* ^+^ (GG61) or *recA* ^-^ (GG66) donor cells containing a tandem array of SXT and R391, and *recA* ^+^ (VB38) or *recA* ^-^ (VB47) recipient cells (Table 1) to distinguish between these possibilities. However, since RecA is required in donor cells for SXT and R391 transfer, probably to alleviate the repression of expression of genes encoding the conjugative transfer machinery (*tra* genes), it was necessary to exogenously express SetC and SetD, the activators of the *tra* genes, in all *recA* donors [51]. Such exogenous activation of transfer genes generally induces a 10- to 100-fold increase in the frequency of ICE transfer ([51] and data not shown); however, since we compare the percentage of hybrids in different backgrounds, rather than the absolute frequency of hybrid formation, the increase in transfer frequency should not distort our results.

**Table 1:** Strains of *E. coli* and plasmids used in this study.

Strain or lasmid	Relevant genotype or phenotype^a^	Reference or source
**Strains**		
CAG18420	MG1655 *lacZU118 lacI42*::Tn*10kan* (Kn^R^)	[65]
CAG18439	MG1655 *lacZU118 lacI42*::Tn*10* (Tc^R^)	[65]
VB112	MG1655 Rf^R^	[52]
VB38	CAG18439 Δ*galK* (Tc^R^)	This study
VB47	CAG18439 Δ*galK* Δ*recA* (Tc^R^)	This study
GG55	VB112 Δ*recA* (Rf^R^)	This study
GG47	GG55 pVI67 (Rf^R^ Ap^R^)	This study
HW220	CAG18439 *prfC*::SXT (Tc^R^ Su^R^ Tm^R^)	[27]
JO99	CAG18439 *prfC*::R391 (Tc^R^ Kn^R^)	[33]
VB40	CAG18439 Δ*lacZ prfC*::SXT::*lacZ*	This study
GG13	CAG18439 Δ*galK prfC*::R391::*galK*	This study
GG61	VB112 *prfC*::[R391::*galK*]-[SXT::*lacZ*] (Rf^R^ Su^R^ Tm^R^ Kn^R^)	This study
GG64	VB112 *prfC*::[R391::*galK* Δ*orf68*]-[SXT::*lacZ* Δ*s065*]	This study
GG65	VB112 *prfC*::[R391::*galK* Δ*orf69*]-[SXT::*lacZ* Δ*s066*]	This study
GG93	VB112 *prfC*::[R391::*galK* Δ (*orf68-orf69*)]-[SXT::*lacZ* Δ (*s065-s066*)]	This study
GG66	GG47 *prfC*::[SXT::*lacZ*]-[R391::*galK*] (Rf^R^ Ap^R^ Su^R^ Tm^R^ Kn^R^)	This study
GG69	GG47 *prfC*::[SXT::*lacZ* Δ*s065*]-[R391::*galK* Δ*orf68*]	This study
GG70	GG47 *prfC*::[SXT::*lacZ* Δ*s066*]-[R391::*galK* Δ*orf69*]	This study
GG102	GG47 *prfC*::[SXT::*lacZ* Δ (*s065-s066*)]-[R391::*galK* Δ (*orf68-orf69*)]	This study
GG125	VB38 *prfC*::[R391::*galK* Δ*mobI*]-[SXT::*lacZ* Δ*mobI*] (Tc^R^ Su^R^ Tm^R^ Kn^R^)	This study
GG171	VB38 *prfC*::[SXT::*lacZ* Δ*int*]-[R391::*galK* Δ*int*] (Tc^R^ Su^R^ Tm^R^ Kn^R^)	This study
GG185	VB38 *prfC*::[R391::*galK*]-[SXT::*lacZ*] (Tc^R^ Su^R^ Tm^R^ Kn^R^)	This study
GG186	VB38 *prfC*::[SXT::*lacZ*]-[R391::*galK*] (Tc^R^ Su^R^ Tm^R^ Kn^R^)	This study
**Plasmids**		
pKD3	Cm^R^ template for one-step chromosomal gene inactivation	[47]
pKD4	Kn^R^ template for one-step chromosomal gene inactivation	[47]
pVI36	Sp^R^ template for one-step chromosomal gene inactivation	[52]
pVI40A	pVI36 *Bam*HI::*P*_lac_-*galK*	This study
pVI42B	pVI36 *Bam*HI::*P*_lac_-*lacZ*	This study
pAH57	*oriR101 repA101* ^Ts^ *cI857* ^ts^ λ*P*_R_-*xis*λ-*int*λ (Ts Ap^R^)	[59]
pVI67	pAH57 Δ(*xis*λ*-int*λ)::*setDC* (Ts)	This study
pVI68	pAH57 Δ(*xis*λ*-int*λ)::*int*_SXT_ (Ts)	This study
pMobI-B	pBAD-TOPO *mobI*	[52]

a *s065* and *s066* of SXT correspond to *orf68* and *orf69* of R391, respectively, according to the annotation of both elements [28],[29]. Ap^R^, ampicillin resistant; Cm^R^, chloramphenicol resistant; Kn^R^, kanamycin resistant; Rf^R^, rifampicin resistant; Su^R^, sulfamethoxazole resistant; Sm^R^, streptomycin resistant; Sp^R^, spectinomycin resistant; Tc^R^, tetracycline resistant; Tm^R^, trimethoprim resistant; Ts, thermosensitive.

**Figure 3:**
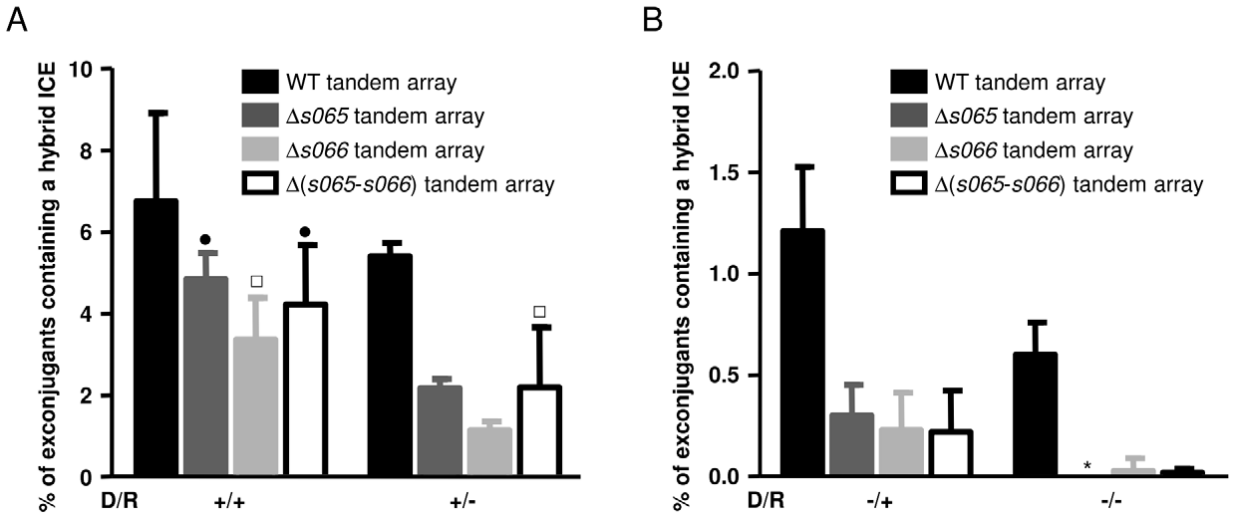
Involvement of *recA*, *s065*, and *s066* in the formation of hybrid ICEs. *recA^+^* (A) or *recA* (panel B) donor strains, which contained either wild-type (WT), Δ*s065*, Δ*s066*, or Δ(*s065*-*s065*) SXT-R391 tandem arrays, were used as donors in these assays. The recipient strains were either *E. coli* VB38 (*recA^+^*) or *E. coli* VB47 (*recA^−^*). D/R + and – indicate the *recA* genotype of the donor and recipient strains, respectively. SetDC was expressed from a plasmid when *recA* donors were used. Bars represent the percentage of exconjugants containing hybrid ICEs and were calculated by dividing the number of exconjugants containing hybrid ICEs (red Tc^R^ Su^R^ Tm^R^ CFU) by the total number of exconjugants (Tc^R^ Su^R^ Tm^R^ CFU). The means and standard deviations obtained from at least three independent assays are shown and the number of colonies containing a hybrid ICE counted for each assay is presented in Table S2. Note the differences in the scale of the y-axis in panels A and B. One-way ANOVA with a Tukey-Kramer post-test was used to compare the means of hybrid ICE-containing exconjugant colonies. The confidence interval for the comparisons of mutant tandem arrays relatively to WT tandem arrays was P<0.001, except □ which indicates P<0.05 and • which indicates that the difference was statistically not significant. * indicates that the percentage of exconjugants bearing a hybrid ICE was below the limit of detection (<0.01%).

Deletion of *recA* from donor cells had a significant effect on the percentage of exconjugants found to contain hybrid ICEs. Conjugation assays with *recA* donors reduced the percentage of hybrids at least 5.6-fold relative to assays with WT donors, both when WT and *recA* recipients were used (p<0.001) (Figure 3A and 3B). In contrast, deletion of *recA* from the recipient cells did not have a significant effect on the percentage of exconjugant colonies containing a hybrid ICE when WT donor cells were used (Figure 3A). When *recA* donor cells were used, there was an ∼2-fold reduction in the percent of exconjugants with hybrid elements in *recA* recipients compared to WT recipients, which was not statistically significant (Figure 3B). Finally, deletion of *recA* from both donors and recipients reduced the percentage of hybrid ICEs detected by more than 11-fold as compared to when *recA* ^+^ was present in both donor and recipient. Taken together, these observations suggest that *recA*-mediated homologous recombination generates the majority of hybrid ICEs and that these recombination events happen both in donor and recipient strains. RecA's role is more readily discerned in donors; however, this may reflect a limitation of our assay in that conjugation facilitates detection of hybrids as discussed below. Notably, 0.60% of exconjugants contained hybrid ICEs even when both donor and recipient strains lacked *recA* indicating that some hybrid ICEs are generated via a *recA*-independent recombination pathway (Figure 3B).

### *s065* and *s066* promote hybrid ICE formation

We explored whether *s065* and *s066*, which encode a single-strand DNA recombinase (unpublished results and [38]) and a putative exonuclease respectively (Figure 1C), also influence the formation of hybrid ICEs, and whether they might account for *recA*-independent generation of these elements. Donor strains harboring tandem arrays of Δ*s065*, Δ*s066*, or Δ(*s065*-*s066*) deletion mutants of SXT and R391 were constructed in *recA* ^+^ and *recA* donor strains, and these strains were used in conjugation assays with *recA* ^+^ (VB38) and *recA* (VB47) recipient strains as described above. Compared to WT donors, when *recA* ^+^ donors lacking *s065*, *s066* or both genes were tested, there was a consistent reduction in the frequency of hybrid formation (Figure 3A). This decrease was generally not statistically significant when *recA* ^+^ donors and recipients were used; however, when *recA* was absent from either donor or recipient cells, the effect of Δ*s065* and/or Δ*s066* deletions became more pronounced. For example, when *recA* donors and WT recipients were used, the percentage of exconjugants containing hybrid ICEs was reduced ∼5 fold by deletion of *s065* and/or *s066*, and when both donors and recipients lacked *recA*, the additional mutations reduced hybrid frequency more than 20-fold (Figure 3B). Presumably, the absence of *recA*, which we have shown prevents formation of a majority of hybrid ICEs, allows the subtler effects of *s065* and/or *s066* deletions to become more apparent. Our data suggest that both *s065* and *s066* contribute to hybrid ICE formation, and that they act in a non-redundant fashion with each other. Additionally, our finding that deletion of both *s065* and *s066* has an effect comparable to that of a single gene deletion indicates that their roles may be interdependent. Since formation of hybrid ICEs was scarcely detectable when both *recA* and *s065/s066* were disrupted, it appears that *s065* and *s066* are required for the majority of *recA*-independent hybrid ICE formation.

Comparisons of the percentages of hybrid formation shown in Figure 3 suggest that *recA* and *s065/s066* may cooperate in generating hybrid ICEs. Approximately 37% of hybrid formation in donor cells is attributable to *recA* as shown by the frequency of hybrid-bearing exconjugants (∼2%) observed in the absence of *s065* and/or *s066* with *recA*- recipients (Figure 3A +/− all but black bar). When donor cells lack *recA* and rely on the *s065*/*s066*-pathway for hybrid formation we found that 0.6% of exconjugants contained hybrids, i.e. 11% of total hybrid formation (Figure 3B −/− black bar). Taken together, these frequencies cannot account for the frequency of exconjugants harboring hybrids observed in the presence of both pathways (5.4%, Figure 3A +/− black bar). Thus, these two pathways, which can function independently, may also act synergistically to promote hybrid ICE formation. However, given the variability in our data, particularly using *recA* ^+^ recipients, definitive evidence for interactions between these pathways is lacking.

### Conjugation is not required for the formation of hybrid ICEs

In the experiments described above, we relied on conjugative transfer to identify hybrid ICEs in exconjugant colonies. However, our observation that some hybrids appear to form in recipient cells, after elements have transferred (as indicated by differences in hybrid formation in *recA* ^+^ and *recA* recipients) suggested that the conjugative process was not necessarily a component of hybrid formation. We took advantage of our previous observations that there is little, if any, conjugative transfer of SXT in broth culture [23], to begin to explore whether conjugation was required for hybrid ICE formation. We tested whether we could detect hybrid formation in a *recA^+^* Δ*galK lacZ^−^* Tc^R^ strain (GG185) bearing a wild-type R391-SXT array (the opposite array orientation as used above) in the absence of a recipient strain. GG185 was passaged with two subcultures in LB broth for 72 h (>100 generations) and then the culture was plated on MCGX indicator medium supplemented with Tc Su Tm, to identify Su^R^ Tm^R^ hybrid ICEs (red colonies), or with Tc Kn, to identify Kn^R^ hybrid ICEs (blue colonies). Kn^R^ hybrid ICEs were detected (0.16±0.05%of colonies) at this point but Su^R^ Tm^R^ hybrids were barely detectable (Table 2). Detection of hybrid ICE formation using this experimental system requires marker loss. We observed greater loss of SXT (20.1±4.9%) than R391 (<0.02%) in this experiment, in accord with a previous report that the ICE located at the right end of the array is more frequently lost and that in this position, R391 is more stable than SXT [33]. Thus, the few detectable Su^R^ Tm^R^ hybrids in this experiment likely reflect the lack of loss of R391 from the tandem array in GG185.

**Table 2:** Percentage of colonies containing hybrid ICEs or single elements recovered over time from a strain initially harboring a wild-type R391-SXT, or a non-transmissible Δ*mobI* R391-SXT tandem array.

		% colonies selected on Kn		% colonies selected on Su Tm	
Strains	Time (h)	Hybrids^a^	Single	Hybrids^a^	Single
GG185	24	0.04±0.04	11.7±3.4	0.02±0.03	0.04±0.07
	72	0.16±0.05	20.1±4.9	0.03±0.05	<0.02^b^
GG125 (Δ*mobI*)	24	0.11±0.02	11.8±0.6	<0.01^b^	0.02±0.03
	72	0.21±0.03	16.1±3.7	<0.01^b^	0.02±0.03
	144	0.38±0.07	22.6±1.2	0.004±0.007	0.09±0.07

a The numbers of colonies containing a hybrid ICE that were counted for each assay are presented in Table S3.

b Detection limit of the assay.

The detection of Kn^R^ hybrids during passage of GG185 in LB broth provides support for the idea that conjugation is not essential for hybrid ICE formation. However, it is possible that there is a low frequency of conjugative ICE transfer in broth cultures. To formally exclude a role for conjugation in hybrid ICE formation, we constructed a strain harboring an R391-SXT array where the ICEs were unable to transfer due to the deletion of *mobI*. MobI is part of the SXT/R391 DNA processing machinery and is thought to recognize and act on *oriT*; deletion of *mobI* renders SXT and R391 non-transmissible but does not impair their excision (data not shown) or the formation of a functional conjugation apparatus [52]. We constructed a Δ*mobI* R391-SXT tandem array in a *recA^+^* Δ*galK lacZ^−^* Tc^R^ strain (VB38) (Table 1). The resulting strain (GG125) was cultivated for 6 days with two daily subcultures (>250 generations) in LB broth with tetracycline as the sole antibiotic. Serial passage allowed for the loss of unselected markers [37], thereby helping to reveal formation of possible hybrid ICEs. The culture was plated at 24, 72, and 144 h post-inoculation on the indicator medium supplemented with the same antibiotics used above to identify hybrid ICEs. As noted above with GG185, loss of SXT from the R391-SXT array in GG125, yielding a single R391 (Kn^R^) ICE, occurred much more frequently than the loss of R391 from this strain (Table 2). Kn^R^ hybrid ICEs were detectable at 24 h, when 0.11% of colonies contained a hybrid ICE, and by 144 h this percentage increased to 0.38% (Table 2). Su^R^ Tm^R^ hybrid ICEs were only isolated after 144 h of culture and only 0.004% of colonies contained hybrids. Potential explanations for the different frequencies with which hybrids were observed are discussed below. However, the results from both selections clearly demonstrate that conjugation is not required for the formation of hybrid ICEs. Furthermore, using a variety of PCR assays (see [37]), three distinct ICE structures were identified among 19 of the Kn^R^ hybrids (data not shown). Thus, the hybrids identified in these experiments cannot be explained by clonal amplification of a single cell containing a hybrid ICE.

### Excision is not required for hybrid ICE formation

Despite existing predominantly as chromosomal-encoded elements, the position of ICEs with respect to host chromosomes is highly dynamic. ICE-encoded *int* and *xis* genes allow them to excise from host chromosomes [53], and this event is thought to be an early step in conjugation. To assess whether extrachromosomal ICE DNA is a required substrate for hybrid ICE formation, we constructed a strain carrying a Δ*int* SXT-R391 array (GG171) (Table 1). GG171 was used in assays similar to those described above for the Δ*mobI* array. After only 24 h of culture, 0.9% of colonies contained a Su^R^ Tm^R^ *galK* ^+^ hybrid ICE, demonstrating that formation of recombinant ICEs does not depend on ICE excision. Thus, chromosomal tandem ICE arrays can serve as a recombination substrate.

## Discussion

Comparative analyses of the genomes of several SXT/R391 ICEs revealed that these elements are mosaics that have been shaped by inter-ICE recombination (Figure 1A). The large set of core genes that are conserved among all SXT/R391 ICEs provides an ample substrate for inter-ICE recombination. Furthermore, the inherent ability of these elements to form tandem array structures [33],[37] increases the opportunities for ICE recombination. Given the high degree of homology between SXT and R391, our finding that *recA* accounts for the generation of the majority of hybrid ICEs is understandable. However, *s065* and *s066*, which are present in all SXT/R391 ICEs, also contribute to formation of recombinant ICEs. The contribution of these ICE λ *bet* and *exo* homologues was easiest to discern in the absence of *recA*; in this context, *s065* and *s066* accounted for the formation of nearly all of the hybrids we detected. These two genes appear to function in the same recombination pathway, since deletion of *s065*, *s066*, or both genes resulted in similar reductions in hybrid formation. Neither ICE excision nor conjugative transfer proved to be essential for generation of hybrid ICEs; instead conjugation appears to facilitate the segregation of hybrids and may provide a means to select for functional recombinant ICEs.

In previous work, we used multiple PCR analyses to show that exconjugants derived from conjugations with donors bearing SXT-R391 arrays occasionally contained a hybrid ICE [37]. This technique was too cumbersome to enable either quantitative or genetic analysis of hybrid ICE formation. The high-throughput semi-quantitative detection method reported here enabled more sensitive analyses of the genetic determinants involved in hybrid ICE formation. Hybrid formation was relatively frequent, as we found that almost 7% of exconjugants selected on Su and Tm contained a recombinant ICE. Since some exconjugants scored as containing a parental ICE array (purple colonies in Figure 2) actually contained a hybrid ICE and a parental ICE, 7% is an underestimation of the true frequency of hybrid formation. Thus, formation of hybrid ICEs, which may have novel combinations of genes conferring resistance to antibiotics, may be fairly common.

While hybrid ICEs were readily detectable in exconjugants using our plate-based screening method, we found that they also form in cells containing tandem arrays of non-transmissible ICEs. Detection of non-transmissible hybrid ICEs seems to depend upon the rate of post-recombinational loss of one or the other ICE, as shown by the coincident increase over time of colonies harboring hybrids (Table 2). Different frequencies of Kn^R^ vs Su^R^ Tm^R^ hybrids formed from the non-transmissible R391-SXT array (Table 2). These differences are probably a consequence of the structure of the array used here. The relatively low frequency of hybrids in donors compared to exconjugants suggests that conjugation facilitated detection of hybrids by allowing for segregation of hybrid ICEs from parental ICEs. In nature, it is possible that conjugation serves to select for functional hybrids that are capable of transmission.

Our data indicate that both *recA* and *s065*/*s066* can mediate hybrid formation independently, and potentially co-operatively as well. RecA's role in homologous recombination has been the subject of extensive study; we assume its mechanism of action parallels that described in previous work. Our models for how *s065* and *s066* mediate hybrid ICE formation are largely based on prior studies of phage-borne *s065* and *s066* homologues. However, there is evidence that S065, like λ Bet, can mediate single-stranded DNA recombination ([38] and our unpublished observations) and that S066 has double-stranded DNA exonuclease activity (Rory Watt, unpublished observations). Thus, it is reasonable to assume that S065 and S066 function in a similar fashion as Bet and Exo to promote ICE recombination. Double-stranded DNA ends are thought to be the principle substrate for the Red pathway in its natural context [40],[54]; Exo is thought to digest the 5′ end of such double-stranded DNA breaks leaving a suitable single-stranded substrate for Bet recombination [55]. Double-strand breaks in ICE DNA could occur in the chromosomal ICE, the excised circular double-stranded ICE or the extrachromosomal circular double-stranded ICE after transfer but prior to re-integration. The latter molecule may be subject to host restriction endonucleases, generating suitable substrates for S066 and S065. Furthermore, DNA damaging agents (UV, antibiotics), which are known to trigger the conjugative transfer of SXT/R391 ICEs, also provide suitable substrates for recombination in the form of double-stranded DNA breaks. It also possible that single-stranded ICE DNA generated in donor cells and transferred to the recipient during conjugation can be a substrate for formation of hybrid elements.

There are particularities of the lifecycles of ICEs and lambdoid phages that suggest that their respective recombination systems may function differently. Unlike λ, which can replicate autonomously as double-stranded DNA (theta replication) during its lytic cycle, SXT/R391 ICEs do not seem to replicate autonomously. This difference likely decreases the opportunities for generating double-stranded breaks that have been shown to be a major substrate for λ Red functions [39],[40],[44]. In addition, the absence of a *gam* ortholog in SXT/R391 ICEs suggests that either RecBCD's exonuclease activity has little impact on recombination catalyzed by S065/S066, i.e. double-stranded DNA extremities are not a significant substrate, or that ICEs encode an unrelated inhibitor of exonuclease V that remains to be indentified.

To our knowledge, the *s065*/*s066* recombination system is the first Red-like recombination pathway to be described in a conjugative element. To date, Red-like recombination genes/systems have been exclusively identified in prophages of both gram-positive and gram-negative bacteria [38]. Interestingly, *s065* and *s066* are part of the core genome found in all SXT/R391 ICEs. Their ubiquity in this family of mobile elements suggests that the generation of diversity via inter-ICE recombination is a key feature of this family of ICEs. The routine formation of tandem ICE arrays in fresh exconjugants [37] and the lack of exclusion between certain SXT/R391 ICEs [34],[35] also suggests that the modus operandi of these elements includes recombination. Recombination is also a central feature of lambdoid phages (for review, see [56],[57]) and Martinsohn et al recently proposed that the λ Red recombination pathway contributes to the mosaic genomes that characterize this family of bacteriophages [58]. Another striking parallel between SXT/R391 ICEs and lambdoid phages is that their transfer (by conjugation or transduction respectively) is greatly increased by damage to host DNA. Expression of *s065* and *s066*, like that of *exo* and *bet*, increases with UV damage to the host (Mariam Quinones, unpublished results). Thus, like the λ Red recombination pathway [41], the *s065/s066* recombination system may serve as a recombinational repair system to promote the formation of functional ICEs capable of exiting from a damaged host and re-establishing themselves in a new host.

While numerous questions regarding the action of S065 and S066 remain to be explored, collectively our findings suggest that these genes promote the plasticity of SXT/R391 ICE genomes. Besides enhancing inter-ICE recombination, it also possible that *s065* and *s066* enable the incorporation of exogenous genetic material into ICE genomes, such as the DNA shown in colors in Figure 1A. Lastly, we identified orthologs of *s065* and *s066* in IncA/C plasmids such as pIP1202 from *Yersinia pestis* biovar *Orientalis*, the causative agent plague. These conjugative plasmids have recently been found to be broadly disseminated among multiply drug resistant zoonotic pathogens [6]. It will be interesting to explore whether these *s065/s066* orthologs contribute to the plasticity of this family of conjugative plasmids.

## Materials and Methods

### Bacterial strains, plasmids, and media

The bacterial strains and plasmids used in this study are described in Table 1. Bacterial strains were routinely grown in Luria-Bertani (LB) broth at 37°C in an orbital shaker and maintained at −80°C in LB broth containing 15% (v/v) glycerol. Colonies harboring hybrid ICEs were screened by plating on MacConkey agar base (Difco) plates supplemented with 0.6% galactose, 80 mg/l X-Gal (5-bromo-4-chloro-3-indoyl-β-D-galactopyranoside) (indicator medium MCGX) and the suitable antibiotics. Antibiotics were used at following concentrations: ampicillin (Ap), 100 mg/l; kanamycin (Kn), 50 mg/l; rifampicin (Rf), 100 mg/l; spectinomycin (Sp), 50 mg/l; sulfamethoxazole (Su), 160 mg/l; trimethoprim (Tm), 32 mg/l; tetracycline (Tc), 12 mg/l.

### Plasmid construction

The oligonucleotides used for construction of plasmids are described in Table S1. Plasmids pVI67 and pVI68, designed to allow conditional expression of SetDC or Int_SXT_, were constructed by replacing the 1,383-bp *Eco*RI/*Nco*I fragment of pAH57 [59] with either a 942-bp *Eco*RI/*Nco*I fragment containing the *setDC* operon of SXT or a 1,367-bp *Eco*RI/*Nco*I fragment containing *int*_SXT_, respectively. *setDC* and *int*_SXT_ were amplified by PCR using primer pairs setDF/setCR and intSF/intSR, respectively, and the DNA of *E. coli* HW220 as a template. Both plasmids are temperature sensitive for replication and allow the expression of the cloned genes from λ*p*_R_ under control of the thermosensitive repressor *cI857*.

Plasmids pVI40A and pVI42B were templates used in the creation of PCR products for the insertion of *lacZ* and *galK* markers into SXT and R391 with the Datsenko and Wanner protocol [47]. These templates contain *galK* or *lacZ*, both under control of *P*_lac_, introduced into the *Bam*HI site of pVI36 [52]. The *P*_lac_-*galK* fragment was made by amplifying by PCR *galK* and the *P*_lac_ promoter sequence using primer pairs galK1F/galK1R and Plac3F/Plac3R, respectively, and the DNA of *E. coli* VB112 as a template. The resulting two fragments were fused using the Splicing by Overlap Extension protocol [60]. The *P*_lac_-*lacZ* of pVI42B was amplified using DNA of *E. coli* VB112 as a template and primer pair lacZ1R/Plac3F. The inserts of all plasmids constructed for this study were sequenced by DNA LandMarks Inc (St-Jean-sur-Richelieu, QC).

### Construction of chromosomal deletions and insertions

The oligonucleotides used for chromosomal deletions and insertions are described in Table S1. Deletion and insertion mutants were constructed by using the one-step chromosomal gene inactivation technique of Datsenko and Wanner [47]. All deletions were designed to be non-polar. The Δ*galK* and Δ*lacZ* mutations were introduced in *E. coli* CAG18439 using primer pairs galKWF/galKWR and lacZW-B/lacZW-F, and plasmids pVI36 and pKD4 as templates. The Δ*recA* mutation was introduced in *E. coli* VB38 and VB112 using primer pair recAWF/recAWR and pVI36 as a template. The Δ*s065*, Δ*s066*, and Δ(*s065-s066*) mutations were introduced in SXT (in strain HW220) using primer pairs 65WF/65WR, 66WF/66WR, and 65WF/66WR, respectively, and template plasmid pVI36. The corresponding mutations Δ*orf68*, Δ*orf69* and Δ(*orf68-orf69*) were introduced in R391 (in strain JO99) using primer pairs betWF/betWR, exoWF/exoWR, and betWF/exoWR, respectively, and pVI36 as a template. Δ*mobI* and Δ*int* mutations were created in R391 using primer pairs orfXRWF/orfXRWR and intRWF/intRWR, respectively, and pKD3 as a template. SXT deletion mutants of *mobI* (VB119) and *int* (BI554) were already available [52],[61].

*lacZ*-tagged SXT was constructed by inserting *P*_lac_-*lacZ* between *traG* and *eex* using primer pair IlacWF/IlacWR and pVI42B as the template, yielding strain VB40. Similarly, *galK*-tagged R391 was created by inserting *P*_lac_-*galK* between *traG* and *merR* using primer pair IgalWF/IgalWR and pVI40A as the template, yielding strain GG13. *P*_lac_-*lacZ* and *P*_lac_-*galK* were also introduced into strains containing SXT and R391 deletion mutants, using P1*vir* generalized transduction and *E. coli* VB40 and GG13 as donor strains. All deletion and insertion mutations were verified by PCR amplification using primers flanking the deletion, cloning and sequencing.

### Construction of strains containing tandem arrays of SXT and R391

Strains containing tandem arrays were constructed by successively transferring SXT::*lacZ* or and R391::*galK* (or their corresponding deletion derivatives) into VB112, yielding strains GG61 to GG65 and GG93. The *recA* null strains GG66 to GG70 and GG102 were created in a similar fashion except that pVI67 was introduced into GG55 prior to the transfer of the ICEs. We used the *mobI* expression vector pMobI-B [52] to mobilize Δ*mobI* ICEs in the construction of strain GG125. We verified that the deletion of *mobI* did not impair SXT or R391 excision using a real-time PCR quantification assay designed to determine the relative proportion of *attP* and *attB* sites per 100 chromosomes as described previously [53]. The *int* expression vector pVI68 was used to mobilize Δ*int* ICEs in the construction of GG171. All strains harboring tandem arrays were tested to determine the relative positions of SXT and R391 in the tandem array by PCR amplification of the leftmost and rightmost ICE-chromosome junctions with primers pairs primer 6/primer 4 and primer 8/primer 9 described by Hochhut et al. [33].

### Conjugation assays and detection of hybrid ICEs

Conjugation assays were performed by mixing equal volumes of overnight cultures of donor and recipient strains grown overnight at 37°C. The cells were harvested by centrifugation, washed in 1 volume of LB broth and resuspended in 1/20 volume of LB broth. The mixtures were then deposited on LB agar plates and incubated at 37°C for 6 hours. The cells were recovered from the plates in 1 ml of LB broth and serial dilutions were prepared. Donors, recipients and exconjugants were selected on LB agar plates containing appropriate antibiotics.

The *setDC* expression vector pVI67 was used in mating assays involving *recA* donor strains. In these experiments, donor strains were grown overnight at 30°C and then cultures were shifted to 42°C for 15 minutes prior to contact with the recipient strain, to induce expression of SetC and SetD.

MCGX indicator agar medium plates supplemented with appropriate antibiotics were used to determine whether SXT, R391, SXT-R391 tandem arrays, or hybrid elements were present in exconjugant colonies or in donor colonies in experiments assessing the necessity of conjugative transfer or excision in hybrid ICE formation. The hybrid ICE detection technique was validated by PCR screening of exconjugant colonies using the primer pairs VISLF/VISLR3, 10SF13/SXT1-13, YND2/ORF16, VISRF/VISRR, VISLF/VISLR2, MER104A/MER103B and VISRF/VISRR2 as described by Burrus and Waldor [37].

### Molecular biology methods

Plasmid DNA was prepared using a QIAprep Spin Miniprep kit (Qiagen) according to manufacturer's instructions. All the enzymes used in this study were purchased from New England BioLabs. PCR assays were performed with the primers described in Table S1 in 20 µl reactions with 1 U of *Taq* DNA polymerase; 1 µl of a mixture of one colony resuspended in 10 µl of HyPure Molecular Biology Grade Water (HyClone) was used as a template in PCR reactions. The PCR conditions were as follows: (i) 3 min at 94°C; (ii) 30 cycles of 30 sec at 94°C, 30 sec at the appropriated annealing temperature, and 1 minute/kb at 72°C; and (iii) 5 min at 72°C. When necessary, PCR products were purified using a QIAquick PCR Purification kit (Qiagen) according to manufacturer's instructions. *E. coli* was transformed by electroporation as described by Dower et al [62] in a BioRad GenePulser Xcell apparatus set at 25 µF, 200 Ω and 1.8 kV using 0.1 cm gap electroporation cuvettes.

## Supporting Information

Table S1 DNA sequences of oligonucleotides used in this study.
(0.05 MB DOC)

Table S2 Number of colonies containing a hybrid ICE counted for each assay presented in Figure 3.
(0.04 MB DOC)

Table S3 Number of colonies containing a hybrid ICE counted for each assay presented in Table 2.
(0.04 MB DOC)
