# Supplementary material for: Mobile Antibiotic Resistance Encoding Elements Promote Their Own Diversity
Source: PLoS Genet. 2009 Dec 18;5(12):e1000775. doi: 10.1371/journal.pgen.1000775 (PMC2786100; doi:10.1371/journal.pgen.1000775)
Supplement: Table S1 — DNA sequences of oligonucleotides used in this study. (0.05 MB DOC) [file pgen.1000775.s001.doc]

**Table S1.** DNA sequences of oligonucleotides used in this study

| **Primer name** | **Use in this study** | **Nucleotide sequence (5’-3’)** |
| --- | --- | --- |
| setDF | Cloning of *setDC* | CTGGAATTCGCAAAGAGTGCTTTCTATCTA |
| setCR | Cloning of *setDC* | TTGCCATGGTAGTACTCTCAAAGTGCACG |
| intSF | Cloning of *int*_SXT_ | CGTGAATTCCATGAGTACAGCGCCAGAA |
| intSR | Cloning of *int*_SXT_ | AAGCCATGGAGGAGTATGAGAAGAAAGAGGA |
| galKWF | Deletion of *galK* | AGCGATATCCATTTTCGCGAATCCGGAGTGTAAGAAGTGTAGGCTGGAGCTGCTTCG |
| galKWR | Deletion of *galK* | CTGACCATCGGGTGCCAGTGCGGGAGTTTCGTTCAGCATATGAATATCCTCCTTA |
| lacZW-B | Deletion of *lacZ* | GCGAAATACGGGCAGACATGGCCTGCCCGGTTATTACATATGAATATCCTCCTTA |
| lacZW-F | Deletion of *lacZ* | TTGTGAGCGGATAACAATTTCACACAGGAAACAGCTGTGTAGGCTGGAGCTGCTTCG |
| recAWF | Deletion of *recA* | GACTATCCGGTATTACCCGGCATGACAGGAGTAAAAGTGTAGGCTGGAGCTGCTTCG |
| recAWR | Deletion of *recA* | GCCGCAGATGCGACCCTTGTGTATCAAACAAGACGAATTCCGGGGATCCGTCGACC |
| galK1F | Cloning of *galK* and fusion with *P*_lac_ | CGGATAACAATTTCACACAGGAAACAGCTATGAGTCTGAAAGAAAAAACACAATCTCTG |
| galK1R | Cloning of *galK* | GGATCCTCAGCACTGTCCTGCTCCTTG |
| Plac3F | Cloning of *P*_lac_ | GGATCCTGAGCGCAACGCAATTAATG |
| Plac3R | Cloning of *P*_lac_ and fusion with *galK* | CAGAGATTGTGTTTTTTCTTTCAGACTCATAGCTGTTTCCTGTGTGAAATTGTTATCCG |
| lacZ1R | Cloning of *lacZ* | GGATCCTTATTTTTGACACCAGACCAACTG |
| 65WF | Deletion of *s065* in SXT | AGGGGCAGACTCCCACTTTGATTGTCGGAGTCCACAGTGTAGGCTGGAGCTGCTTCG |
| 65WR | Deletion of *s065* in SXT | TCCCGTCAGGGTTAGTAAAATGAATGCGTGGCGCTACATATGAATATCCTCCTTA |
| 66WF | Deletion of *s066* in SXT | TCTCCTTCGGGGGAGTCTCCCCTCAAAGGAGACAATGTGTAGGCTGGAGCTGCTTCG |
| 66WR | Deletion of *s066* in SXT | CATGAGAAAGGAGCCGAAATCGGCTCCAAGTGAACGCATATGAATATCCTCCTTA |
| betWF | Deletion of *orf68* in R391 | AGGGTCAGACTCCCACTTTGATTGTCGGAGTCCACAGTGTAGGCTGGAGCTGCTTCG |
| betWR | Deletion of *orf68* in R391 | TAATCCCGCCAGGGTTAGTAAAATGAATGCGTGGCGATTCCGGGGATCCGTCGACC |
| exoWF | Deletion of *orf69* in R391 | CTCCTTCGGGGGAGTCTCCCCCTCAAAGGAGACAATGTGTAGGCTGGAGCTGCTTCG |
| exoWR | Deletion of *orf69* in R391 | CATGAGAAAGGAGCCGAAATCGGCTCCAAGTGAACGATTCCGGGGATCCGTCGACC |
| IlacWF | Insertion of *P*_lac_-*lacZ* in SXT | CAGGAAGGAACTGAAAATAGAAGGTAGCCAGCTTGTGTGTAGGCTGGAGCTGCTTCG |
| IlacWR | Insertion of *P*_lac_-*lacZ* in SXT | GGTATCCCAAAAAGCCAAAAAAATAGCCAGCCAACCCATGAGAATTAATTCCGGGGA |
| IgalWF | Insertion of *P*_lac_-*galK* in R391 | AACAAGGAGTAGAAGCTCAAGGCTAACGAAGTTTTAGTGTAGGCTGGAGCTGCTTCG |
| IgalWR | Insertion of *P*_lac_-*galK* in R391 | ACGGAGAAATATATCCCTATCTACGTAATTAAGCCACATGAGAATTAATTCCGGGGA |
| intRWF | Deletion of *int* in R391 | TCATTAAAATCAAAACTAGGGCTGGGCTTATAACACATATGAATATCCTCCTTA |
| intRWR | Deletion of *int* in R391 | GAGGAGTATGAGAAGAAAGAGGACTGAGCCTCTTTGTGTAGGCTGGAGCTGCTTCG |
| orfXRWF | Deletion of *mobI* in R391 | TTTGGTTTTTGGGGTTAATTGGATGGGGAAATTGGGTGTAGGCTGGAGCTGCTTCG |
| orfXRWR | Deletion of *mobI* in R391 | GGGACCAATTACCGCGAGTTAAGTAGCGCAGTTAACATATGAATATCCTCCTTA |
