# Supplementary material for: Mobile Antibiotic Resistance Encoding Elements Promote Their Own Diversity
Source: PLoS Genet. 2009 Dec 18;5(12):e1000775. doi: 10.1371/journal.pgen.1000775 (PMC2786100; doi:10.1371/journal.pgen.1000775)
Supplement: Table S2 — Number of colonies containing a hybrid ICE counted for each assay presented in Figure 3. (0.04 MB DOC) [file pgen.1000775.s002.doc]

**Table S2.** Number of colonies containing a hybrid ICE counted for each assay presented in Figure 3.

| **D/R** | **Tandem array** | **% of CFU** | **# of CFU** |
| --- | --- | --- | --- |
| +/+ | WT | 6.75 | 260 |
| +/+ | Δ*s065* | 4.82 | 199 |
| +/+ | Δ*s066* | 3.35 | 152 |
| +/+ | Δ(*s065*-*s066*) | 4.21 | 69 |
| +/- | WT | 5.41 | 245 |
| +/- | Δ*s065* | 2.15 | 85 |
| +/- | Δ*s066* | 1.14 | 62 |
| +/- | Δ(*s065*-*s066*) | 2.05 | 13 |
| -/+ | WT | 1.21 | 68 |
| -/+ | Δ*s065* | 0.29 | 27 |
| -/+ | Δ*s066* | 0.23 | 16 |
| -/+ | Δ(*s065*-*s066*) | 0.22 | 8 |
| -/- | WT | 0.6 | 31 |
| -/- | Δ*s065* | 0.0 | 0 |
| -/- | Δ*s066* | 0.03 | 2 |
| -/- | Δ(*s065*-*s066*) | 0.02 | 4 |
