# Supplementary material for: Mobile Antibiotic Resistance Encoding Elements Promote Their Own Diversity
Source: PLoS Genet. 2009 Dec 18;5(12):e1000775. doi: 10.1371/journal.pgen.1000775 (PMC2786100; doi:10.1371/journal.pgen.1000775)
Supplement: Table S3 — Number of colonies containing a hybrid ICE counted for each assay presented in Table 2. (0.04 MB DOC) [file pgen.1000775.s003.doc]

**Table S3.** Number of colonies containing a hybrid ICE counted for each assay presented in Table 2.

| **Strain** | **Time (h)** | **Selection** | **% of CFU** | **# of CFU** |
| --- | --- | --- | --- | --- |
| GG185 | 24 | Kn | 0.04 | 3 |
| GG185 | 24 | Su Tm | 0.02 | 5 |
| GG185 | 72 | Kn | 0.16 | 22 |
| GG185 | 72 | Su Tm | 0.03 | 4 |
| GG125 | 24 | Kn | 0.11 | 19 |
| GG125 | 24 | Su Tm | <0.01*^a^* | 0 |
| GG125 | 72 | Kn | 0.21 | 14 |
| GG125 | 72 | Su Tm | <0.01*^a^* | 0 |
| GG125 | 144 | Kn | 0.38 | 40 |
| GG125 | 144 | Su Tm | 0.0004 | 1 |

*^a^* Detection limit of the assay.
